# Erythroblastosis Transformation-Specific Regulated Gene 1 (ERG) Immunohistochemistry in the Diagnosis of Acute Myeloid Leukemia

**DOI:** 10.7759/cureus.61168

**Published:** 2024-05-27

**Authors:** Nathanael C Lucas, Catherine Neal, Blake Hsu, Penny Wright

**Affiliations:** 1 Department of Haematology, Christchurch Hospital, Te Whatu Ora/Health New Zealand, Christchurch, NZL; 2 Department of Anatomical Pathology, Christchurch Hospital, Te Whatu Ora/Health New Zealand, Christchurch, NZL

**Keywords:** leukemic blast cells, immunohistochemistry staining, diagnose, acute myeloid leukaemia, malignant haematology

## Abstract

Introduction: The erythroblastosis transformation-specific regulated gene 1 (ERG) is a transcription factor that can be used as an immunohistochemical (IHC) marker in the diagnosis and prognostication of malignancy. ERG was initially used in prostate cancer; however, it is a useful marker in extramedullary myeloid disease. Patients with acute myeloid leukemia (AML), dry bone marrow aspirate, and CD34, CD117-negative blast cells can be in a diagnostic dilemma. This audit aimed to (a) validate ERG IHC in bone marrow trephine samples, (b) quantify ERG IHC positivity in an AML cohort, and correlate concordance with CD34 and CD117 IHC, when available, and (c) to see whether ERG is a useful adjunct in the diagnosis of cases of AML.

Methods: A retrospective audit was completed of all new and relapsed cases of AML over one year at a single center. For inclusion, patients needed a trephine specimen at presentation, and all had a hematoxylin and eosin(H&E) specimen, ERG IHC, and at least one or both of CD34 and CD117 IHC. Four pathologists independently assessed the stains quantitatively and qualitatively in comparison to the morphology seen on the H&E sample. The kappa value was used to assess agreement.

Results: Seventeen patients with AML met the inclusion criteria. All specimens had H&E, CD34, and ERG stains; 9/17 (53%) had CD117 IHC. ERG demonstrated high concordance with blast cells on H&E morphology, with a high agreement among pathologists. Qualitatively, pathologists recognized that ERG spared lymphoid nodules; however, it also stained granulocytes at various maturation stages.

Conclusion: ERG is a sensitive marker for the diagnosis of AML. ERG can help visualize blast cells that have been confirmed by ancillary tests. More research into the utility of ERG in AML diagnostics is recommended.

## Introduction

The erythroblastosis transformation-specific regulated gene 1 (ERG) is a transcription factor that is involved in numerous key biological processes including cell proliferation, differentiation, angiogenesis, inflammation, and apoptosis [1]. In the setting of hematopoiesis, ERG plays a role in the development of hematopoietic stem cells, regulating self-renewal and differentiation [2,3].

Current research on ERG has predominantly focused on its role in prostate carcinoma and Ewing sarcoma. In prostate cancer, an ERG gene fusion with the promoter region of the androgen-induced TMPRRSS2 gene is a common finding and is used as a diagnostic tool and a target for immunotherapy [4].

In acute myeloid leukemia (AML), a small subset of patients have t(16:21) ERG:FUS fusion, which was first identified in 1994 as a driver of leukemogenesis [5]. A recent review of 52 patients with ERG:FUS demonstrated poor prognosis as well as association with GATA2, SMAD4, and RUNX1 mutation [6]. The 2022 WHO classification recognizes ERG as an important driver in acute erythroid leukemia [7].

The diagnostic workup of AML involves a combination of morphology, cytogenetics, immunophenotyping from flow cytometry, and, if available, next-generation sequencing myeloid gene panels [8,9]. While this is diagnostic for the majority of patients, a small subset presents with a dry bone marrow aspirate and few blasts in peripheral blood but with immature cells identifiable on trephine biopsy. In these cases, immunohistochemistry (IHC) remains the key tool in making a diagnosis. Currently, we use two IHC markers: CD34 and CD117 to identify immature precursors.

IHC has inherent limitations. IHC staining is reported by a pathologist using a light microscope via a manual method giving an estimate of the overall percentage positivity. Manual enumeration of positive cells is poorly reproducible. IHC stains may be nuclear, membrane, or cytoplasmic. Membrane and cytoplasmic stains are subject to significant inter- and intra-observer variability when compared to nuclear staining [10]. Importantly, ERG is a nuclear stain whereas CD34 and CD117 are not. The reproducibility of ERG, therefore, makes it an attractive option for identifying immature myeloid cells, with ongoing research into electronic enumeration methods, particularly for nuclear IHC staining [11].

To the best of our knowledge, four previous studies have looked at ERG IHC in myeloid malignancy [12-15].

Anti-ERG was developed as an immunohistochemistry stain for prostate cancer in 2011 [16]. In a hematopoietic setting, the first study in 2016 identified ERG as a useful marker in the diagnosis of leukemia cutis (LC). They reviewed 32 biopsies, 16 with LC, and 16 with reactive infiltrates. ERG positivity was found in 13/16 LC cases, whereas it was negative in all reactive cases. This demonstrated a positive predictive value of 100% and a negative predictive value of 84.2% [14]. The second study focused on bone marrow specimens, correlated ERG with myeloperoxidase (MPO), and looked at the strength of ERG staining in a disease setting. They reviewed 24 cases (six normal, 12 AML, and six myeloproliferative) and demonstrated that ERG is concordant with myeloperoxidase (MPO) IHC, and the ERG staining was useful in identifying the myeloblast population in 7/12 AML cases [12]. Koo and Natkunam have recently published a review of 207 cases of immature and mature hematolymphoid lesions. They found ERG immunopositivity in 15 of 16 (94%) acute myeloid leukemias/myeloid sarcomas, including four out of five (80%) CD34-negative/CD117-negative acute myeloid leukemias/myeloid sarcomas. ERG positivity was also seen in all nine cases of B-lymphoblastic and T-lymphoblastic leukemia/lymphoma. They concluded that ERG is a useful adjunct in the identification of blastic hematolymphoid neoplasms [13]. Vargas et al. looked at the utility of ERG in myeloid sarcoma, patients with blastic plasmacytoid dendritic neoplasm (BPDCN), and normal controls. ERG was expressed in a majority of myeloid sarcoma and was not expressed in any patients with BPDCN. Importantly, it was also expressed on non-neoplastic myeloid lineages; therefore, the authors recommend ERG must be used in an antibody panel in the workup of myeloid infiltrates but was useful in excluding BPDCN as a differential [15].

This retrospective cohort study aimed to (a) validate ERG immunohistochemistry staining in bone marrow trephine samples in our center, (b) quantify ERG IHC positivity in an AML cohort and correlate concordance with CD34 and CD117 immunostaining, when available, and (c) to see whether ERG is a useful adjunct in the diagnosis of cases of AML where the blast cell population has proven difficult to identify by other means.

This article was previously presented as a meeting abstract at the 2023 Haematology Society of Australia and New Zealand - New Zealand Branch Annual Scientific Meeting on April 4, 2023.

## Materials and methods

A retrospective audit of all new and relapsed cases of AML between November 1, 2021, and November 1, 2022, referred to a single center (Christchurch Hospital, Canterbury, New Zealand). Inclusion criteria included a recorded diagnosis of AML at presentation or relapse with a bone marrow trephine biopsy completed at our center.

A review of clinical history was completed on all patients meeting inclusion criteria. Demographic data, immunophenotype (by flow cytometry), cytogenetics, and myeloid next-generation sequencing (NGS) panel (if available) were recorded into a secure EXCEL spreadsheet for further analysis.

All trephine biopsies were formalin-fixed for at least two hours in neutral buffered formalin before decalcification. Specimens were then decalcified for two hours at room temperature in 9% hydrochloric acid. After decalcification, the specimens had an additional 44 minutes of fixation at 45 degrees during automated tissue processing. Specimens were then embedded in paraffin wax. The tissue sections were then stained with hematoxylin and eosin (H&E) using local laboratory protocols. IHC studies were performed on the Roche/Ventana Benchmark Ultra Automated Stainer following standard protocols. Patients all had ERG IHC and at least one or both of CD34 and C-KIT (CD117). The ERG antibody used was a rabbit monoclonal (clone EPR3864), prediluted, from Roche/Ventana. The CD34 antibody was a mouse monoclonal (clone QBEnd/10), 1:200, from Novocastra/Leica, and the C-KIT (CD117) antibody was a rabbit polyclonal (clone polyclonal), 1:400, from Dako/Agilent.

For IHC validation, we followed the College of American Pathologists Pathology and Laboratory Quality Center guidelines [17]. We focused on the first two points. First, laboratories must validate all IHC tests before placing them into a clinical service. This is achieved by correlating new test results with morphology and expected results, comparing the new test results with results of prior testing of the same tissues on the validated assay, comparing new test results with tissue validation set in another laboratory, and comparing new test results with previously validated non-immunohistochemical tests. Second, for initial validation of the new immunohistochemistry stain, laboratories should achieve at least 90% concordance between the new test and the comparator test.

All patient notes and ancillary tests were initially reviewed to confirm the diagnosis of AML. Four hematopathologists independently assessed the trephine biopsies. H&E morphology and ERG, CD34, and CD117 IHC slides were assessed. For ERG, CD34, and CD117, hematopathologists graded positivity on a three-point scale, such as negative, weak positive, or strong positive.

We used Stata (version 16; StataCorp, College Station, TX) to generate agreement statistics, with a kappa value of 0.81-1.0 considered almost perfect agreement, 0.61-0.8 considered substantial agreement, 0.41-0.6 considered moderate agreement, 0.21-0.4 considered fair agreement, and 0-0.2 considered poor agreement. Qualitative analysis of pathologist’s comments was assessed using a general inductive approach described by Thomas [18].

## Results

Eighteen patients out of 29 (with either new AML or a frank relapse) had a trephine biopsy. One case was removed from histopathological analysis following diagnostic uncertainty; therefore, 17 patients were included in the final analysis (17/29: 59%).

Participant characteristics are shown in Table 1. The majority of patients were NZ European (83.3%) with one Māori and two African patients. The mean age was 60.9 years, and two-thirds of patients were male. The majority had a normal karyotype, with other aberrations outlined in Table 1.

**Table 1:** Participant characteristics NZ: New Zealand.

Characteristics	Number of participants (N = 17)
Ethnicity	
NZ European	14 (82.4%)
Māori	1 (5.9%)
African	2 (11.8%)
Age (Years)	
Mean	60.9
Median	64.5
Sex	
Female	6 (35.3%)
Male	11 (64.7%)
Karyotype	
Normal	10 (58.8%)
t(8:21)	1 (5.9%)
8	1 (5.9%)
Complex (>3 abnormalities)	4 (23.5%)
Insufficient sample	1 (5.9%)

Four hematopathologists (three senior consultants and one advanced trainee registrar) reviewed 60 slides. This comprised of 17 H&E slides, 17 ERG slides, 17 CD34 slides, and nine CD117 slides. The initial analysis looked at consistency in reporting between pathologists. ERG demonstrated substantial agreement: κ = 0.76 as did CD117 and κ = 0.62. CD34 demonstrated moderate agreement: κ= 0.58.

On review of H&E specimens, morphologically immature cells/blasts were identified in all 17 cases. For confirmation of AML diagnosis, ancillary tests were performed: cytogenetics (16/17), flow cytometry (17/17), and myeloid gene panel (11/17). Six patients did not have a myeloid gene panel due to age at diagnosis as per local protocols.

**Figure 1:**
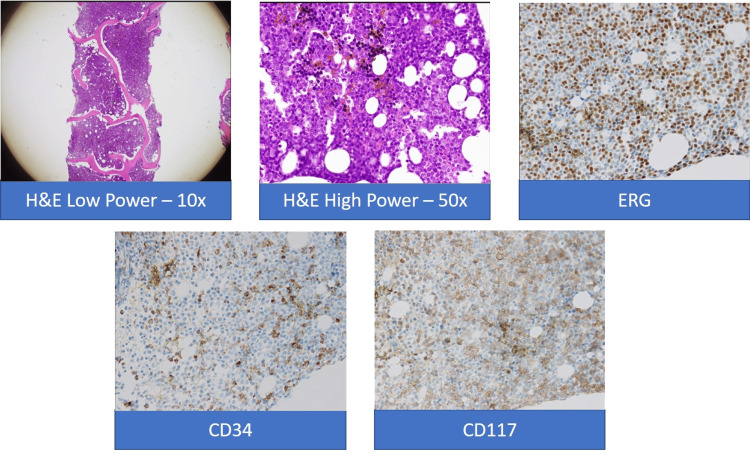
An example from a case in the study who was positive for CD34, CD117, and ERG on IHC. This figure shows H&E stain at 10x and 50x objectives and demonstrates CD34 (membranous stain), CD117 (cytoplasmic/membranous stain), and ERG (nuclear stain) positivity. H&E: Hematoxylin and eosin; ERG: Erythroblastosis transformation-specific regulated gene 1; IHC: Immunohistochemistry.

When reviewing morphological blasts and concordance with ERG, CD34, and CD117, ERG stained positive in 16/17 cases giving a sensitivity of 0.93, whereas the sensitivity for CD34 was 0.59 and that of CD117 was 0.66. One case that did not stain for ERG was negative for both CD34 and CD117. This case was diagnosed as osteosclerotic AML [19]. Figure 1 demonstrates an example of ERG IHC when compared to H&E, CD34, and CD117 slides.

Six cases were CD34- and CD117-negative AML. Three reviewers thought blasts were identified in 5/6 of these cases using ERG, and one reviewer thought blasts were identified in 4/6 cases using ERG.

Qualitative analysis from the reviewers' written comments demonstrated pathologists appreciated the nuclear staining of ERG, and all commented that it was easier to determine positivity when compared with CD34 and CD117. Some reviewers commented on the fact that ERG spared lymphoid nodules and stained megakaryocytes prominently. All reviewers commented that ERG also stained mature granulopoietic cells and blasts in cases of AML with maturation.

## Discussion

We demonstrate that ERG is a sensitive marker for the diagnosis of AML. When myeloblasts are identified by an experienced hematopathologist, ERG highlights them in the majority of cases (16/17), demonstrating that ERG has a higher sensitivity than the commonly used IHC markers for AML, CD34, and CD117.

A common pitfall of trephine morphology is the consistency of reporting. Nuclear stains are less prone to inter-observer variability [11]. In our study, reporting of the nuclear stain ERG was significantly more consistent when compared to CD34 and CD117, demonstrated by its high Kappa value. Qualitative analysis also demonstrated that pathologists found ERG to be an easy stain to examine when compared with other stains, with the added benefit of a nuclear stain making it easier to discern the blast cell morphology. Consistency of reporting is pivotal to enabling patients’ accurate diagnosis and appropriate care.

It is not uncommon to have a situation where a diagnosis of AML has to be made from a trephine biopsy. These cases can be difficult especially when IHC for CD34 and CD117 is negative. We hypothesized that ERG might be of greatest utility when a patient has CD34- and CD117-negative cells, but the pathologist is convinced of blast-like morphology. In our study, ERG IHC highlighted the morphologic blasts in the majority of these samples (5/6 for three reviewers and 4/6 for one reviewer), thus confirming that it is useful in this setting. However, as it is not specific, ERG would need to be used as part of a panel with ancillary tests such as cytogenetics, flow cytometry, and molecular studies.

Although ERG is not perfect, as it is not a marker of immaturity like CD34 and CD117 and will stain all stages of granulocytic maturation, it has also been demonstrated in other studies [15]. This is important as it limits the utility of ERG to situations where a pathologist is confident of blast cell morphology and has ancillary tests such as cytogenetics, flow cytometry, or molecular aberrations helping to confirm AML diagnosis. ERG staining other granulopoietic precursors also limits the accurate enumeration of blasts in certain subtypes of AML. For example, in the case of AML with maturation, ERG would be unlikely to help determine the 20% blasts required.

Other limitations of this study include the retrospective design, small sample size, and the fact that it only involved one laboratory. The small sample size increases the risk of selection and sampling bias. This means further research needs to be done to confirm the precision, reliability, and applicability of ERG in difficult cases.

In the future, to fully validate the ERG IHC in AML diagnostic workup, we hope to get another laboratory to validate ERG in their patient population. Further studies utilizing anti-ERG flow cytometry could assist in determining ERG specificity, and a more comprehensive assessment of ERG in normal bone marrow specimens will help determine the role of ERG IHC in the future.

## Conclusions

In summary, ERG is an IHC stain that could help confirm the diagnosis of acute myeloid leukemia. Being a nuclear stain, it is easy to interpret, shows high concordance with morphologic blasts, and identifies populations seen by current markers of immaturity (CD34 and CD117) in most cases, suggesting the potential utility of ERG in difficult-to-diagnose AML. Further larger studies, looking into the specificity, precision, and accuracy of ERG, are needed. We conclude that ERG is a useful additional marker that can be added to the IHC panel in the workup of acute myeloid leukemia.
